# Enzymatic Decontamination of G-Type, V-Type and Novichok Nerve Agents

**DOI:** 10.3390/ijms22158152

**Published:** 2021-07-29

**Authors:** Pauline Jacquet, Benjamin Rémy, Rowdy P. T. Bross, Marco van Grol, Floriane Gaucher, Eric Chabrière, Martijn C. de Koning, David Daudé

**Affiliations:** 1 Gene&GreenTK, 19–21 Boulevard Jean Moulin, 13005 Marseille, France; pauline.jacquet@gene-greentk.com (P.J.); benjamin.remy21@gmail.com (B.R.); floriane.gaucher@gene-greentk.com (F.G.); 2 TNO Department CBRN Protection, Lange Kleiweg 137, 2288 GJ Rijswijk, The Netherlands; rowdy.bross@tno.nl (R.P.T.B.); marco.vangrol@tno.nl (M.v.G.); 3 Institut Hospitalo-Universitaire (IHU) Méditerranée Infection, Aix-Marseille Université, 13005 Marseille, France; 4 Institut de Recherche pour le Développement (IRD), Assistance Publique Hôpitaux de Marseille (APHM), Unité Microbe Evolution Phylogénie et Infection (MEPHI), 13005 Marseille, France

**Keywords:** decontamination, phosphotriesterase, nerve agents, organophosphorus, Novichok, sarin, VX

## Abstract

Organophosphorus nerve agents (OPNAs) are highly toxic compounds inhibiting cholinergic enzymes in the central and autonomic nervous systems and neuromuscular junctions, causing severe intoxications in humans. Medical countermeasures and efficient decontamination solutions are needed to counteract the toxicity of a wide spectrum of harmful OPNAs including G, V and Novichok agents. Here, we describe the use of engineered OPNA-degrading enzymes for the degradation of various toxic agents including insecticides, a series of OPNA surrogates, as well as real chemical warfare agents (cyclosarin, sarin, soman, tabun, VX, A230, A232, A234). We demonstrate that only two enzymes can degrade most of these molecules at high concentrations (25 mM) in less than 5 min. Using surface assays adapted from NATO AEP-65 guidelines, we further show that enzyme-based solutions can decontaminate 97.6% and 99.4% of 10 g∙m^−^^2^ of soman- and VX-contaminated surfaces, respectively. Finally, we demonstrate that these enzymes can degrade ethyl-paraoxon down to sub-inhibitory concentrations of acetylcholinesterase, confirming their efficacy from high to micromolar doses.

## 1. Introduction

Organophosphorus chemicals (OPs) are highly toxic compounds inhibiting cholinesterase enzymes from the central nervous system and causing severe poisoning in humans [1,2]. They have been developed as organophosphorus nerve agents (OPNAs) for military purposes but have also been considered for use in agriculture and constitute the first class of insecticides worldwide responsible for severe acute and chronic intoxications [3,4,5,6]. OPNAs can be divided into three groups: (i) G-agents (G for German) including sarin, cyclosarin, tabun and soman, which are known as non-persistent; (ii) V-agents (V for Venomous, Victory or Viscous, depending on the source) such as VX, which are persistent; and (iii) compounds belonging to the A-series of agents, or Novichok agents (Supplementary Figure S1) [5,7]. While the use of OPNAs is banned by the Organisation for the Prohibition of Chemical Weapons (OPCW), recent examples show that these agents are still considered in terrorist attacks and asymmetric conflicts, constituting a serious threat for populations. For example, sarin was used in Syria in 2013 [8,9], VX in Malaysia (2017) [10] and, more recently, Novichok agents were used in the UK (2018) and Russia (2020) for poisoning purposes [11,12,13]. The recent events involving Novichok agents have sparked interest in these new compounds, and a number of publications have appeared since their use in 2018 [14,15,16,17]. However, these contributions are restricted to reviewing historical information or discussing theoretical data on Novichok agents [18,19,20,21], due to the unavailability of these compounds for experimental work. Thus, (verifiable) experimental data remain very scarce to date, and thus far, enzymatic degradation of Novichok agents has only been tested with the OPAA enzyme [22]. In November 2019, it was decided that Novichok agents should be added to Schedule 1 of OPCW [23].

Given the toxicity of OPs, particularly OPNAs, decontamination methods and medical countermeasures have been developed to counteract their poisoning effects [6,24,25,26]. On the one hand, significant efforts have been devoted to the development of prophylactic and curative approaches for treating patients, particularly those in the military and security forces [27,28,29]. On the other, only limited studies have focused on the development of solutions that could be used for decontaminating people, materials or surfaces exposed to OPs and for limiting cross contamination [6]. Chemical solutions such as oxidative or very basic decontaminants including bleach, sodium hydroxide and alkoxide/amine-based decontaminants can be corrosive and are therefore limited to the decontamination of inert or hardened surfaces, while Fuller’s earth, RSDL sponges or DECPOL gloves are mainly used for the local decontamination of skin or surfaces, respectively, but are not compatible with large scale use [6,30,31,32,33]. To address these limitations, enzymes have emerged as a potential general solution compatible with the decontamination of people, material and the environment, without toxicity or secondary pollution [25,34,35]. Of these, paraoxonases (PONs), diisopropylfluorophosphate fluorohydrolase (DFPase), organophosphate acid hydrolase (OPAA) and phosphotriesterases (PTEs) have mainly been considered. The latter are of particular interest given their natural capacity to degrade OP insecticides and have been further engineered to increase their efficacy towards OPNAs [36,37]. The main target application of these enzymes has been their use in humans as OPNA scavengers [29,38], or as detoxification catalysts to destroy the agents before they could reach their toxicological target. A PTE was also successfully produced in tobacco plants providing an alternative strategy for OP decontamination [39]. However, their potential use for material or skin decontamination has barely been considered. Two commercial enzyme-based products, namely DEFENZ and Landguard A900, have been developed by Genencor and CSIRO Ecosystem Sciences for the decontamination of OPNA and insecticides, respectively [26,40]. Based on wild-type PTE, DEFENZ aimed to tackle OPNA contaminations and showed promising results in enzyme reactors when evaluated by the United States Environmental Protection Agency. Surface decontamination assays, however, showed inferior results and probably further limited development of the technology [41,42]. Conversely, Landguard A900 relied on proficient engineered PTE and was shown to efficiently degrade a large spectrum of insecticides, particularly for on-farm management practices for the removal of chlorpyrifos and diazinon in furrow runoff; however, it was not considered for defense purposes [43].

This study aims to demonstrate (i) that enzyme-based formulations can be used to decontaminate a broad spectrum of nerve agents including the poorly studied Novichok agents and (ii) that such formulations can be used to degrade high concentrations of neurotoxic chemicals used in solution or spread on surfaces. To this end, we evaluate the potential of two engineered PTEs, namely GG1 and GG2, developed by the company Gene&GreenTK [44,45]. We first demonstrate the outstanding efficacy of the solution to degrade a wide spectrum of OPs including four insecticides and six OPNA surrogates, rapidly and completely. Building on these favourable results, we show the formulation’s ability to degrade high concentrations of real nerve agents. We chose to focus our study on high concentrations of nerve agents to provide the basis for the evaluation of enzymes for decontamination purposes. Thus, the hydrolytic activity towards four G-agents (i.e., sarin, cyclosarin, tabun and soman), the V-agent VX and Novichok agents (A230, A232 and A234), used at a 25 mM concentration, is discussed. The capacity of this enzyme-based solution to decontaminate soman and VX, following NATO guidelines for decontamination efficacy evaluation from a surface, is further highlighted. Finally, to complete our results, which were obtained at high concentrations, we demonstrate the capacity of these enzymes to degrade OP to sub-inhibitory concentrations of a recombinant variant of human acetylcholinesterase (rHAChE), using ethyl-paraoxon as a model substrate.

## 2. Results and Discussion

### 2.1. Kinetic Parameters of Engineered Enzymes Towards Ethyl-Paraoxon and OPNA Surrogates

As a first step, the two engineered PTEs, GG1 and GG2, were evaluated for their capacity to hydrolyse ethyl-paraoxon, a model substrate in studies of PTE. Both enzymes were shown to hydrolyse ethyl-paraoxon with high catalytic efficiencies reaching k_cat_/K_M_ values above 10^7^ s^−1^∙M^−1^, which is in the same order of magnitude as previously reported for potent OP-degrading enzymes (Table 1) [40]. Next, the ability of GG1 and GG2 to degrade OPNA surrogates, i.e., coumaric derivatives of sarin (CM Sarin), cyclosarin (CM Cyclosarin), soman (CM Soman), tabun (CM Tabun), VX (CM VX) [46,47] and another VX surrogate namely DEVX [48], was evaluated. For these agents, k_cat_/K_M_ values were estimated by using one-phase or two-phase decay non-linear regression of the degradation curves (Table 1). Both enzymes were capable of rapidly hydrolysing all these surrogate agents. GG1 and GG2 displayed similar efficacy towards CM Soman or CM Tabun and were even more effective for the degradation of CM Sarin and CM Cyclosarin, respectively. It should be noted that racemic surrogates were used in these experiments. Indeed, with CM Soman, two rates of hydrolysis by GG1 and GG2 were observed, as determined by two-phase decay non-linear regression of the data. Both enzymes’ fast k_cat_/K_M_ values were around 10^5^ s^−1^∙M^−1^ and the slow k_cat_/K_M_ values were around 10^4^ s^−1^∙M^−1^. No obvious differences in enantiomer degradation rates were observed for the other coumaric surrogates. Notably, GG1 showed a better capability to hydrolyse the two V-agent surrogates, especially towards DEVX, which was not converted by GG2. As DEVX is known to be a closer analogue of VX than CM VX [48], GG1 was considered to be more relevant than GG2 for addressing VX decontamination. These results confirm that the two engineered PTE are active over a broad range of organophosphorus chemicals. GG1 showed activity towards all tested substrates, while no activity was detected against DEVX with GG2, which was more active on G-agents surrogate than GG1.

### 2.2. Decontamination of Live G- and V-Agents with Engineered Enzymes

The next phase in the evaluation entailed subjecting the enzymes to real OPNAs in experiments that would reflect a more realistic setting. Thus, much higher concentrations of agents were used (25 mM) in combination with the enzymes (20 µM) in Tris buffer (100–200 mM Tris, pH 9, 50 mM NaCl). The higher concentration of agent facilitated the monitoring of the reactions with ^31^P NMR. Due to the extreme toxicity of OPNAs, the experiments were carried out in a high-tox facility and were conducted by staff that had received training to work with these agents. In these experiments, only one substrate concentration (25 mM) was tested, and the performance of the enzyme solutions was evaluated by determining half-life (t_1/2_) values (Table 2). Additionally, k_cat_ values were estimated considering that the tested concentration was much higher than the K_m_ value (Table 2).

**Figure 1:**
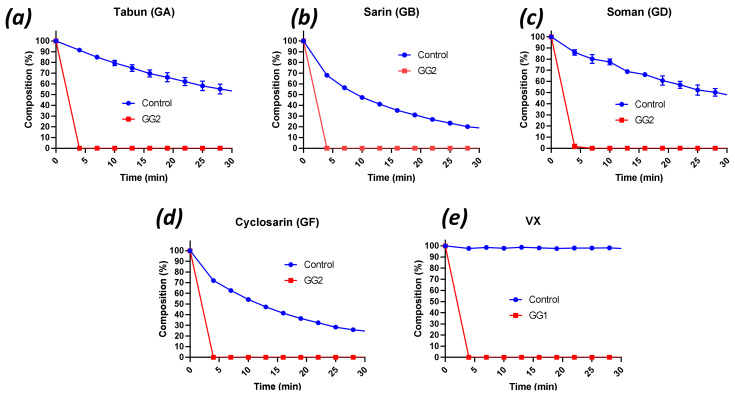
Degradation curves of (**a**) tabun GD, (**b**) sarin GB, (**c**) soman GD, and (**d**) cyclosarin GF by GG2, and of (**e**) VX hydrolysis by GG1. Agent concentration: 25 mM, enzyme concentration; 20 µM. Experiments were conducted in 100–200 mM Tris buffer pH 9, 50 mM NaCl at 23 °C.

Although GG1 and GG2 performed comparably in the degradation of G-agent surrogates, GG2 performed better than GG1 with CM Cyclosarin. Therefore, GG2 was chosen for experiments with the live G-agents. Conversely, as GG1 showed a better performance with the V-agent surrogates than GG2, the former was used in the experiments with VX.

(Figure 1a–d) shows the degradation curves of soman (GD), sarin (GB), tabun (GA) and cyclosarin (GF) by GG2, while Figure 1e shows hydrolysis of VX by GG1. All curves also display the corresponding controls (no enzyme). All G-agents as well as VX were rapidly degraded by the enzyme solutions (Figure 1), and complete hydrolysis was observed within 4 min in all cases. Comparison with the control curves showed that most hydrolyses could be ascribed to enzyme activity. The hydrolysis products were assigned by ^31^P NMR and LC-MS analysis. Agent degradation occurred through hydrolysis of the P-F bonds in G-agents and the P-S bond in VX, respectively. It is important to emphasise that P-OEt scission in VX, resulting in the highly toxic agent E2192, was not observed. Inspection of the ^31^P NMR spectra of the control curves revealed that, besides spontaneous hydrolysis, the G-agents formed adducts with the buffer molecules [49], which could be confirmed by LC-MS analysis. Adduct formation was not observed in the enzyme-mediated reactions, because the rates of catalysis were much higher than those of adduct formation.

To further evaluate the performance of the enzyme solutions in relation to the various G- and V-agents, additional experiments were executed. The hydrolysis profiles of VX (25 mM) exposed to serial dilutions of the GG1 enzyme solution were determined (Supplementary Figure S2a). The respective half-lives are given in Table 2. The GG1-enzyme solution could be diluted at least 100× (i.e., 0.2 µM enzyme) before complete degradation of VX would take more than 15 min (a common duration in military decontamination doctrine). These experiments thus show that the undiluted GG1 solution has more than enough enzyme capacity to allow fluctuations in the VX-concentration in future decontamination challenges without significantly increasing the time required to ensure complete degradation of VX. Next, the stability of the enzyme solution was assessed, by preparing two dilutions of GG1 (200× and 400×, corresponding to 0.1 µM and 50 nM enzyme) and adding VX after 4.5 h of ageing at room temperature. Supplementary Figure S2b shows that the activity of the GG1-enzyme solution decreased over time and the half-lives approximately tripled. Similar experiments were conducted with the GG2-enzyme solution (Supplementary Figure S2c,d). This enzyme solution could be diluted at least 400× (50 nM enzyme) before complete degradation of GD would take more than 15 min, and the enzyme activity was not significantly affected by ageing up to 4.5 h.

Using 1000× diluted GG2 (i.e., 20 nM), it became evident that this enzyme was comparably reactive towards most of the G-agents used (Supplementary Figure S2e). Sarin was degraded with the greatest efficacy (complete degradation within 4 min, and a half-life of 3.4 min even with 3000× diluted GG2, 6.7 nM), while GF showed a hydrolysis rate comparable to GD (t_1/2_ 2–3 min). Tabun, however, showed a step hydrolysis in the first couple of minutes (65% tabun remaining after 4 min) followed by slower hydrolysis that was reminiscent of the rate of degradation in the control (no enzyme, Figure 1a). Because of the disappointing performance of diluted GG2 towards tabun, enzyme GG1 was also subjected to GA. The 200× dilution (0.1 µM enzyme) was chosen to be able to make a comparison with the VX hydrolysis rate (Table 2). GG1 was proved capable of hydrolysing tabun, albeit 2.6 times slower than VX. Representative examples of ^31^P NMR spectra of G- and V-agents with and without enzyme, and LC-MS data of the agent-Tris adducts are provided in Supplementary Figures S3–S15.

Finally, for all the degradations, [S] >> K_M_, thus allowing us to estimate k_cat_ (Table 2). GG1 estimated k_cat_ values were found around 10^2^ s^−1^ with VX and GA, in the same order of magnitude as for ethyl-paraoxon and DEVX. For GG2, estimated k_cat_ values are around 10^3^ s^−1^ with GF and GD and were comparable to k_cat_ values towards ethyl-paraoxon.

Impressively, the results demonstrate that GG1 and GG2 based formulations can degrade G- or V-agents with high efficacy. Experiments with diluted enzymes further demonstrate that concentrations of GG1 or GG2 can be significantly decreased while maintaining high performance.

### 2.3. Degradation of Novichok Agents with Engineered Enzymes

**Figure 2:**
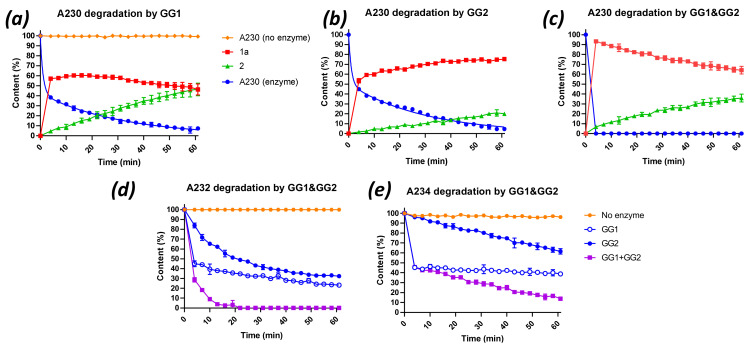
Degradation curves of Novichok agents A230, A232 and A234 by GG1 and GG2 enzymes. Degradation of A230 by GG1 (**a**) and GG2 (**b**) or both (**c**). Red and green represent the products of A230 hydrolysis. Degradation of A232 (**d**) and A234 (**e**) by GG1, GG2 or both. Agent concentration: 25 mM, enzyme concentration; 20 µM (for GG1 and GG2 mix, 1:1 ratio). Experiments were conducted in 100–200 mM Tris buffer pH 9, 50 mM NaCl at 23 °C.

In contrast to G- and V-agents, the enzymatic degradation of Novichok agents has been poorly studied, given the lack of availability of these compounds before 2018. To our knowledge, only one recent report described the hydrolyses of Novichok compounds by the OPAA enzyme, although their rates of hydrolysis were found to be low compared with G-agents [22]. The capacity of PTEs to hydrolyse Novichok agents has never been reported. Here, the ability of both the GG1 and GG2 enzymes to hydrolyse the three Novichok agents A230, A232 and A234 (Supplementary Figure S1) was evaluated. These compounds appeared to be stable in Tris buffer at pH 9 for the experimental period (60 min). However, the addition of the enzymes resulted in bi-phasic hydrolysis profiles, particularly with GG1, which was attributed to different hydrolysis rates of the two enantiomers of each substrate. Under the conditions used, GG1 achieved approximately 50% hydrolysis of all Novichok agents within 4 min (Figure 2a,d,e). However, the rates of the hydrolysis reactions of the second enantiomer, when using the mixture of GG1/GG2, clearly differed between the compounds (A230 > A232 > A234). That difference in hydrolysis rate between the enantiomers was particularly remarkable with A234, the second enantiomer of which showed hardly any degradation after 60 min of experimental time (Figure 2e). GG2 reached a result which was comparable to GG1 in terms of the hydrolysis of A230 (Figure 2b). However, GG2 had more difficulty with A232 and A234, and the differences in hydrolysis rates between the enantiomers was smaller, while the overall rates were quite low (Figure 2a–e).

Finally, the three Novichok agents were subjected to a 1:1 mixture of GG1 and GG2. The same total enzyme concentration in these experiments was maintained as in the single enzyme experiments. A230 was now completely hydrolysed within 4 min (Figure 2c). The hydrolysis profiles of A232 and A234 clearly reflected the sum of the separate contributions of GG1 and GG2 (Figure 2d,e), leading to complete degradation of A232 within 15 min and near complete degradation of A234 (14% remaining) within one hour. These results clearly show that GG1 and GG2 can work in concert and preferentially target a different enantiomer of the Novichok agents. As for G- and V- agents, the efficacy of the degradation of Novichok agents through the mixture of enzymes was evaluated by measuring the half-life values (Table 3). In silico docking, experiments were further performed to try to identify the origin of enantiopreference towards Novichok agents. The simulations highlighted the difference of accommodation between A230 (R) and (S) enantiomers, but no clear conclusion could be inferred regarding the tendency of GG1 and GG2 to preferentially recognise one enantiomer over another (Supplementary Figure S16a–c).

Although complete degradation of Novichok agents could be obtained, k_cat_ values of GG1/GG2 mixture towards A232, A232 or A234 were found to be lower than for G- and V-agents.

**Figure 3:**
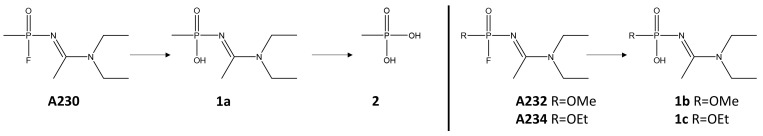
Degradation reactions of Novichok agents observed in this study.

As revealed by ^31^P NMR and LC-MS analysis, the (initial) product in each case was formed by hydrolysis of the P–F bond, giving compounds (**1a**) for A230, (**1b**) for A232 and (**1c**) for A234 (Figure 3). However, in contrast to A232 and A234, the A230 hydrolysis reactions showed a secondary conversion of the initial hydrolysis product (**1a**) to methylphosphonic acid (**2**). To evaluate whether this secondary reaction can be ascribed to the enzyme activity (competitive substrate), to spontaneous hydrolysis or both, the enzymes were removed by centrifugation over a molecular weight cut-off filter after 22 min of reaction, and the evolution of the reaction mixture was followed by NMR. Interestingly, concentrations of (**1a**) and (**2**) remain unchanged after enzyme removal, suggesting that conversion of (**1a**) to (**2**) is enzymatically catalysed (Supplementary Figure S17).

These results demonstrate that engineered PTE can be active against Novichok agents, a finding that has not previously been reported, to the best of our knowledge, showing that an enzyme-based solution, which is active against the whole spectrum of OPNA, can be obtained.

### 2.4. Decontamination of VX or GD-Contaminated Surfaces with the Enzyme-Solutions

**Figure 4:**
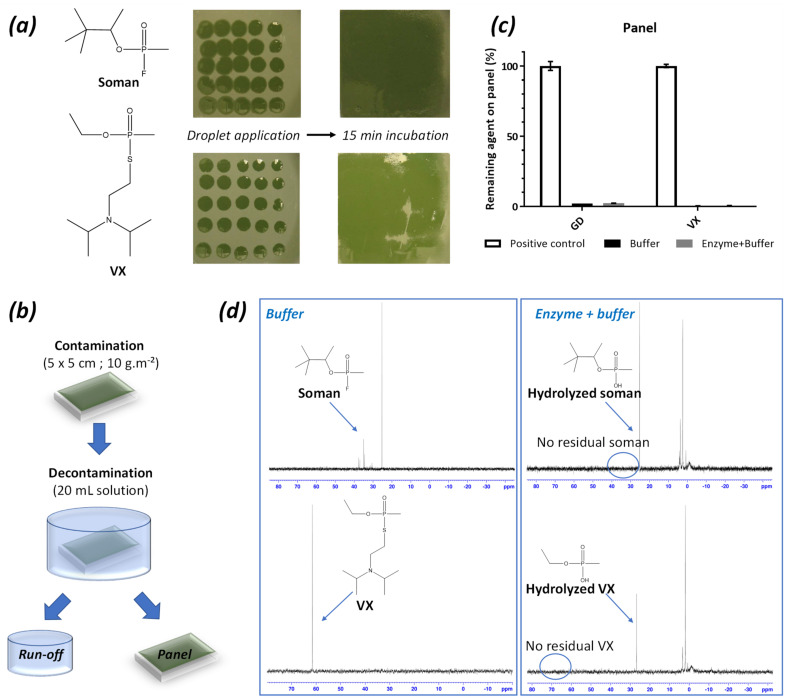
Enzyme decontamination of painted panels. (**a**) Contamination of panels with droplets of agent to reach a final contamination of 10 g∙m^−^^2^ of soman or VX. (**b**) Contaminated panels (5 × 5 cm) are incubated for 15 min in 20 mL of decontamination solutions. (**c**) Evaluation of residual soman (GD) and VX on panels after decontamination. (**d**) Evaluating remaining soman and VX in liquid decontamination (run-off) solution.

To further address the potential of the two enzymes for decontamination purposes, panels painted with chemical agent resistant coating (CARC) were contaminated with high amounts (10 g∙m^−2^) of VX and soman, according to the NATO AEP-65 performance requirements (Figure 4). After 15 min of equilibration, the contaminated panels were immersed in the decontamination solutions, with and without enzymes, for 15 min. The used decontamination liquids were set aside, and the panels were rinsed with water. The residual amount of agent on the panels was determined by GC-FPD quantification of agent in extracts of the panels. The residual amount was compared to the positive control (no decontamination) to give a decontamination efficiency. The run-off liquids (i.e., the used decontamination liquids) were analysed by ^31^P NMR. Soman was removed from the panels regardless of the presence of enzyme (97.6% removal in the presence of enzyme, 97.9% without enzyme) suggesting that decontamination was mainly driven by the buffer washing effect. Nevertheless, analysis of the run-off showed the absence of soman (31/38 ppm) and only the presence of hydrolysed soman (26 ppm), while a large fraction of intact soman was detected in the run-off of the buffer-only decontamination. Regarding VX, high surface decontamination efficacies were observed both in the presence and absence of enzyme (99.4% and 99.5%, respectively). Similarly, no residual VX (62 ppm) was found in the run-off, but only the hydrolysis product (26 ppm). By contrast, VX remained fully intact in the run-off in the absence of enzyme. While OPNAs degrading PTE were mainly considered for developing prophylactic approaches and medical countermeasures, our results underline that they are also of outmost interest for external decontamination purposes and would deserve to be further considered in this way to provide sustainable and non-toxic decontamination solutions for OPNAs.

### 2.5. Enzymes Protect rHAChE from Inhibition by Ethyl-Paraoxon

**Figure 5:**
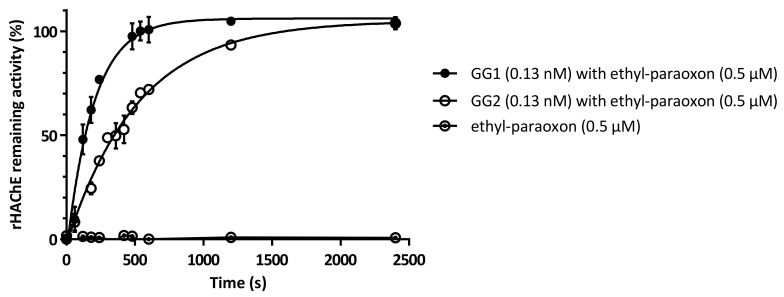
rHAChE inhibition assay [50] with ethyl-paraoxon (0.5 µM) previously incubated with GG1 or GG2 (0.13 nM) and control without PTE.

Alongside decontamination experiments involving high concentrations of agents, further assays were performed to demonstrate the potential of these enzymes to degrade OPs to innocuous doses. Acetylcholinesterase inhibition assays, using a recombinant variant of human acetylcholinesterase (rHAChE) expressed in *Escherichia coli*, were thus performed [50,51]. OPs are known to react with the catalytic serine of AChE through phosphylation resulting in the formation of an inactive and covalent phosphoenzyme. rHAChE was thus used as a sensitive probe to demonstrate the capacity of PTE variants to degrade ethyl-paraoxon to micromolar doses (Figure 5). A concentration of ethyl-paraoxon of 0.5 µM was sufficient to completely inhibit acetylcholinesterase activity. Enzymes had to be diluted down to 0.13 nM (i.e., [substrate]/[enzyme] ≈ 3850) to allow rHAChE protection curves to be measured. Impressively, while using low concentrations of PTEs, rHAChE was completely and rapidly protected from ethyl-paraoxon. These results clearly demonstrate that PTEs can degrade OP down to sub-inhibitory doses of rHAChE. This inhibition assay was further used to confirm the catalytic efficiencies of GG1 and GG2 towards ethyl-paraoxon. k_cat_/K_M_ (s^−1^∙M^−1^) values of 3.87 ± 0.33 × 10^7^ and 1.46 ± 0.33 × 10^7^ were determined for GG1 and GG2, confirming previous results obtained by following p-nitrophenolate release during ethyl-paraoxon hydrolysis. In addition to our evaluations regarding high concentrations of OPNA, the results obtained here suggest that GG1 and GG2 enzymes are able to degrade very low doses of ethyl-paraoxon down to sub-inhibitory concentrations of rHAChE.

## 3. Materials and Methods

### 3.1. Chemicals

Ethyl-paraoxon was purchased from Sigma-Aldrich, St. Louis, MO, USA (purity >95%, analytical standard). Coumaric derivatives of cyclosarin, sarin, soman, tabun, VX and DEVX (purity 95%, laboratory grade) were synthesised on demand by Enamine Ltd. (Riga, Latvia). Soman, tabun, cyclosarin, sarin, VX, A230, A232 and A234 were synthesised in-house (TNO, Rijswijk, The Netherlands). The compounds were >95% pure (determined using quantitative ^1^H NMR).

### 3.2. Production and Purification of PTEs

GG1 and GG2 were engineered from *Brevundimonas diminuta* PTE (P0A434) [44] and were produced as previously described [45]. In short, pET22b-GG1 and pET22b-GG2 were transformed in *E. coli* BL21(DE_3_)-pGro7/GroEL (TaKaRa) chaperone expressing strain. The starter and culture were produced in a ZYP auto-inducible medium (10 g∙L^−1^ tryptone, 5 g∙L^−1^ yeast extract, 50 mM (NH_4_)_2_SO_4_, 100 mM KH_2_PO_4_, 100 mM Na_2_HPO_4_, 0.5% (*w*/*v*) glycerol, 0.05% (*w*/*v*) glucose, 0.2% (*w*/*v*) α-lactose) supplemented with ampicillin (100 µg∙mL^−1^) and chloramphenicol (34 µg∙mL^−1^). Cells were grown at 37 °C and stirred until OD_600 nm_ reached 0.8–1, then induction was performed by addition of L-arabinose (0.2% (*w*/*v*)) and CoCl_2_ (0.2 mM). At the same time, the temperature was reduced to 16 °C. After 20 h of growth, cells were harvested by centrifugation (30 min, 4400 g, 10 °C), resuspended in PTE lysis buffer (100–200 mM Tris pH 9, 50 mM NaCl, 10 µg∙mL^−1^ DNAse I, 0.25 mg∙mL^−1^ lysozyme and 0.1 mM PMSF) and stored at −80 °C. Cells were lysed by sonication (3 × 30 s Qsonica, Q700 (Newtown, CT, USA); Amplitude 40) and centrifuged (15 min, 10,000 g, 10 °C). Crude lysates were used with activities around 450–1100 U∙mL^−1^ for GG1 and 6000–25,000 U∙mL^−1^ for GG2 on ethyl-paraoxon. When needed, recombinant PTEs, with Strep-TEV tag, were purified by injection on StrepTrap column (StrepTrap HP 5 mL, GE Healthcare (Chicago, IL, USA): ÄKTA Avant). Protein concentration was measured with a NanoDrop 2000 spectrophotometer (Thermo Scientific, Waltham, MA, USA), and protein purity was assessed by SDS-PAGE.

### 3.3. Kinetic Parameters

Catalytic parameters were measured at 25 °C in triplicate using 96-well plates with a reaction volume of 200 µL. Reactions were recorded by a microplate reader (Synergy HT, BioTek, Winooski, VT, USA) in a 6.2 mm path length cell and using the Gen5.1 software. All the kinetic assays were performed in PTE activity buffer (200 mM Tris pH 9.0, 50 mM NaCl).

Ethyl-paraoxon (within 0.01 and 2 mM) and ethyl-parathion (within 0.05 and 1 mM) hydrolysis were monitored at 405 nm (ε = 17,000 M^−1^∙cm^−1^). Chlorpyrifos (within 0.05 and 1 mM) hydrolysis was followed at 310 nm (ε = 5562 M^−1^∙cm^−1^). Malathion (within 0.1 and 5 mM) and DEVX (within 0.01 and 2 mM) hydrolysis were monitored at 412 nm (ε = 14150 M^−1^∙cm^−1^) by the addition of 5,5’-dithiobis-2-nitrobenzoic acid (DTNB) to the reaction mixture (200 mM Tris pH 9.0, 50 mM NaCl and 2 mM DTNB). Graph-Pad Prism 6 software was used to obtain the catalytic parameters by fitting the data to the Michaelis-Menten (MM) equation.

For coumaric OPNA surrogates, measurements were taken at 10 µM, and hydrolysis was followed by fluorescence (360/40, 460/40 nm). We assumed that $K_{M}\;\gg \left[S\right]$, allowing us to estimate kinetic parameters with the following equation $\frac{k_{cat}}{K_{M}}=\frac{k}{\left[E\right]}$ and using one-phase decay non-linear regression in Graph-Pad Prism 6 software. For CM Soman hydrolysis, degradation of both enantiomers was discernible. In this case, data were treated with two-phase decay non-linear regression, enabling us to determine a fast and slow $k_{cat}/K_{M}$ [50].

For OPNAs, only $k_{cat}$ estimations were determined considering that $\left[S\right]\gg K_{M}$ (here, 25 mM for all tested agents) since $v=v_{max}$ and then $k_{cat}=\frac{v_{max}}{\left[E_{T}\right]}$. Spontaneous hydrolysis was deducted from global hydrolysis to determine only enzyme-related hydrolysis.

### 3.4. Degradation Experiments with OPNAs

Caution: OPNAs are extremely toxic and must be used by trained personnel in a facility that has been authorised to use these agents.

A Bruker Avance III 400 MHz spectrometer was used to monitor (^31^P NMR) nerve agent degradation experiments. A typical experiment was conducted as follows. Water (1.0 mL 10% D_2_O/H_2_O) was added to a vial containing the lyophilised buffer/enzyme mixture and shaken until all solids were dissolved giving 0.7 mg∙mL^−1^ of enzyme (0.02 mM in 100–200 mM Tris buffer pH 9, 50 mM NaCl). This solution is referenced as the undiluted concentration. Dilutions of enzymes were obtained by mixing Tris buffer in 10% D_2_O/H_2_O with the enzyme stock solution, until the desired dilution was obtained. Pure agent (4–6 µL of GB, GD, GA, GF, or VX) was weighed in a 4 mL vial and an appropriate amount of the buffer/enzyme stock solution and 10% D_2_O/H_2_O was added to give a total concentration of 25 mM agent.

Degradation of the Novichok agents was conducted in a similar way, with the difference that stock solutions (500 mM) of agent in acetonitrile were used instead of neat agent. Thus, 30 µL of agent stock solution was mixed with 570 µL of enzyme stock, giving 25 mM agent concentration and 5% acetonitrile in the reaction mixture.

The mixed GG1/GG2 experiments with Novichok agents were performed by mixing 30 µL of agent stock solution, and 285 µL of both enzyme stock solutions.

Directly after combining the agent and the enzyme(s), the mixture was vortexed for 10 s and transferred to an NMR tube. Sequential ^31^P NMR measurements were started as soon as possible, using the standard ^31^P{^1^H} pulse sequence and 64 scans per measurement. As a result of locking and shimming, the first measurement commenced after 4 min, and subsequent measurements took 3 min each, up to a total time of about one hour (20 measurements). The integrals of the agent and its degradation products were determined, and their sum was set to 100%, enabling representation of degradation of the agent or formation of the hydrolysis products as a fraction of the total phosphorus content. All degradation experiments were performed at least in duplicate.

### 3.5. Liquid Chromatography Tandem Mass Spectrometry (LC-MS) Analyses

LC-MS experiments were performed on a Waters HPLC system connected to a Waters QDA mass spectrometer (Waters Chromatography B.V., Etten-Leur, The Netherlands). Samples were diluted appropriately to prevent overloading of the column. The column was a Cortes-T3 4.6 mm × 100 mm, 2.7 µm (Waters Chromatography B.V., Etten-Leur, The Netherlands) with a gradient of 0–100% solution B in A). A: 5% acetonitrile + 0.2% formic acid; B: 95% acetonitrile + 0.2% formic acid; gradient: 1 min 0% B, then a gradient to 100% B in 8 min.

Alternatively, a Phenomenex Kinetex biphenyl 4.6 mm × 150 mm, 2.6 µm (Phenomenex, Utrecht, The Netherlands) column was used, employing the same buffers except that 0.1% TFA was used instead of formic acid. Gradient: 1 min 0% B, then to 100% B in 12 min. Generally, a flow of 1.0 mL∙min^−1^ was used.

### 3.6. Production and Partial Purification of rHAChE

Plasmid coding for rHAChE, pET32b-rHAChE-3G4, was kindly provided by Moshe Goldsmith from the Weizmann Institute (Rehovot, Israel). As previously described [50], pET32b-rHAChE-3G4 plasmid was transformed in *E. coli* Origami B cells. Starter and culture were produced in 2^x^YT medium (16 g∙L^−1^ tryptone, 10 g∙L^−1^ yeast extract and 5 g∙L^−1^ NaCl) supplemented with ampicillin (100 µg∙mL^−1^). Culture was grown at 37 °C with agitation until an OD_600 nm_ of 1 was reached, then isopropyl β-d-1-thiogalactopyranoside (IPTG, 0.2 mM final concentration) was added and the temperature was reduced to 16 °C. After 24 h of growth, cells were harvested by centrifugation (20 min, 4400 g, 10 °C). Cells were resuspended in rHAChE lysis buffer (13 mM Tris pH 8.0, 33 mM NaCl, 10 mM EDTA, 10% (*w*/*v*) glycerol and 0.25 mg∙mL^−1^ lysozyme) and stored at −80 °C. Lysate was sonicated (3 × 30 s Qsonica, Q700; Amplitude 40) and centrifuged (15 min at 10,000 g and 10 °C), octyl glucoside 0.1% (*w*/*v*) was added. A first ammonium sulfate precipitation was performed, saturation from 0% to 40% for two hours at 4 °C. After centrifugation (15 min, 10,000 g, 10 °C), a second ammonium sulfate precipitation was performed, saturation from 40% to 50% overnight at 4 °C. After centrifugation (15 min, 10,000 g, 10 °C), the pellet was resuspended in activity buffer (13 mM Tris pH 8.0, 33 mM NaCl, 10 mM EDTA, 10% (*w*/*v*) glycerol and 0.1% (*w*/*v*) octyl glucoside). rHAChE was injected in desalting column (HiPrep 26/10 desalting, GE Healthcare; ÄKTA Avant), to remove ammonium sulfate, then in exclusion size chromatography (HiLoad 16/600 Superdex^TM^ 75pg, GE Healthcare; ÄKTA Avant). rHAChE activity was determined using Ellman’s reagent (DTNB, 4 mM) and acetylthiocholine (2.5 mM), and reaction was followed at 412 nm for 10 min with a microplate reader (Synergy HT, BioTek, Winooski, VT, USA) [52]. Fractions containing partially purified rHAChE were pooled and concentrated to ≈3 U∙mL^−1^.

### 3.7. Surface Decontamination Experiments

These experiments were conducted in collaboration with Proqares (www.proqares.com). Metal panels (5 × 5 cm, 3 mm thick) were coated with (Dutch) chemical agent resistant coating (CARC), according to the Dutch army painting protocol, by van Geffen BV, Tilburg, the Netherlands. The coating was applied on all sides of the panels. In each experiment, a total of 12 panels were used (three for decontamination with enzyme in buffer, three for decontamination with buffer, five positive controls (contaminated, but not decontaminated) and one negative control (not contaminated, but decontaminated).

The decontamination solution (“decontaminant”) was prepared by dissolving enzyme (0.7 mg∙mL^−1^) in Tris buffer (200 mM pH 9, 50 mM NaCl). A 1 M HCl solution was freshly prepared (“quenching solution”). The panels were conditioned to ambient conditions for at least 24 h prior to testing. Agent (VX or GD) was applied to the panels (≈25 droplets of ≈1 µL were divided over the surface, with a total of 25 mg for 25 cm^2^ (=10 g∙m^−2^)). Moreover, 11 CARC panels (five positive controls, 2 × 3 test panels) were contaminated and incubated for 15 min at 20 °C. Three panels were decontaminated by submerging each panel for 15 min in 20 mL “decontaminant” and three panels were decontaminated with “buffer”. One uncontaminated panel was also decontaminated with “decontaminant” (negative control). The five remaining contaminated panels served as positive controls.

After decontamination, two samples (≈2 mL each) of each of the three “decontaminant” experiments and two samples (≈2 mL each) of each of the three used “buffer” experiments were collected directly after decontamination. HCl (1 M) was added in 1 of each pair of samples until the pH reached 5–6 (quenching enzyme activity). These samples were filtered, D_2_O was added to give a 10% *v*/*v* D_2_0 in the resulting mixture, and the samples were immediately analysed with ^31^P NMR.

The decontaminated panels were briefly rinsed with water and dried with adsorbent paper without rubbing. Next, all panels were extracted twice with 10 mL of diethyl succinate for 90 min. The amount of agent in each extract was quantified by GC-FPD (Agilent 6890, employing a Wax column (15 m, 530 µm diameter and 1 µm film thickness). Temperature programmes—GD: isothermal at 160 °C; VX: 0.4 min at 170 °C, followed by a temperature gradient to 195 °C (50 °C∙min^-1^). Quantitative data were calculated using a calibration curve. The amount of agent found on a panel was calculated by the sum of the amounts of agent found in the two consecutive extractions.

### 3.8. rHAChE Inhibition Assay

Inhibition assays were carried out as previously described [50,51]. First, PTEs (0.13 nM) were incubated with ethyl-paraoxon (5 µM) in PTE activity buffer (200 mM Tris pH 9.0, 50 mM NaCl) and samples were collected for 40 min. Secondly, these samples (PTE 0.13 nM and ethyl-paraoxon 0.5 µM) were incubated for 15 min with rHAChE (activity around 2.2 U∙mL^−1^). Finally, rHAChE activity was measured using Ellman’s reagent (DTNB, 4 mM) and acetylthiocholine (2.5 mM). The reaction was monitored at 412 nm for 10 min with a microplate reader (Synergy HT, BioTek, Winooski, VT, USA) [52]. Curves were fitted using one-phase decay equation on GraphPad Prism 6 software, and $k_{cat}/K_{M}$ values were determined.

## 4. Conclusions

In this paper, two engineered PTEs were evaluated for their hydrolytic potential towards OPNAs, with the aim of developing an environmentally benign, non-corrosive and effective formulation for surface decontamination. Several representative examples of G-agents, VX as a representative V-agent and three Novichok agents were tested with these enzymes. The kinetic results clearly demonstrated the potential for rapid degradation of G- and V-type agents. These enzymes were also able to degrade Novichok compounds albeit with lower overall hydrolysis rates as compared with G- and V-agent hydrolysis. However, the use of a mixture of both enzymes, under the same conditions, turned out to completely destroy A230 and A232 as well as the majority of A234, showing that these enzymes can effectively work in concert and that they preferentially target a different one of the two enantiomers of each agent. This finding will be useful for future engineering and further optimisation of enzyme activity. Preliminary surface decontamination experiments, following NATO’s guided protocols, clearly indicate that crude enzymatic solutions show promise in the cost-effective decontamination of surfaces contaminated with nerve agents. Furthermore, these enzyme-based innocuous solutions may also be of prime interest for skin decontamination purposes and are deserving of further study.

## Figures and Tables

**Table 1:** Activity of PTE variants against insecticides as well as G- and V-agent surrogates.

Substrates			PTE Variants					
			GG1			GG2		
			k_cat_ (s^−1^)	K_M_ (µM)	k_cat_/K_M_ (s^−1^∙M^−1^)	k_cat_ (s^−1^)	K_M_ (µM)	k_cat_/K_M_ (s^−1^∙M^−1^)
Insecticides	Ethyl-paraoxon		251 ± 2	13 ± 0.5	2.0 ± 0.4 × 10^7^	3395 ± 55	148 ± 9	2.3 ± 0.7 × 10^7^
	Ethyl-parathion		11 ± 0.2	375 ± 27	2.9 ± 0.1 × 10^4^	29 ± 0.4	129 ± 3	2.3 ± 0.1 × 10^5^
	Malathion		65 ± 11 × 10^−2^	8 ± 2 × 10^3^	8.0 ± 0.6 × 10^1^	31 ± 8 × 10^−2^	11 ± 4 × 10^3^	2.8 ± 0.3 × 10^1^
	Chlorpyrifos		16 ± 0.8 × 10^−2^	150 ± 12	1.1 ± 0.1 × 10^3^	23 ± 3 × 10^−2^	524 ± 68	4.5 ± 0.2 × 10^2^
G-agent surrogates	CM Soman	Fast ^a^	*	*	3.1 ± 1.1 × 10^5^	*	*	3.1 ± 0.8 × 10^5^
		Slow ^a^	*	*	4.1 ± 4.0 × 10^4^	*	*	1.3 ± 0.4 × 10^4^
	CM Tabun		*	*	2.4 ± 0.9 × 10^5^	*	*	1.1 ± 0.2 × 10^5^
	CM Sarin		*	*	8.2 ± 2.4 × 10^6^	*	*	3.5 ± 0.4 × 10^6^
	CM Cyclosarin		*	*	6.4 ± 2.6 × 10^5^	*	*	2.0 ± 1.2 × 10^6^
V-agent surrogates	CM VX		*	*	7.1 ± 1.3 × 10^6^	*	*	5.9 ± 1.0 × 10^6^
	DEVX		262 ± 14	4254 ± 215	6.2 ± 0.1 × 10^4^	n.d.	n.d.	n.d.

Kinetic parameters were determined at 25 °C. n.d.: not determinable. * Kinetic parameters for coumaric surrogates were estimated using one-phase decay non-linear regression. ^a^ For CM Soman hydrolysis, degradation of both enantiomers was discernible. In this way, data were treated with two-phase decay non-linear regression.

**Table 2:** OPNA enzymatic degradation half-life.

Enzyme	Agent (25 mM)	Enzyme Dilution ^a^	Half-Life (min) ^b^	k_cat_ (s^−1^) ^c^
GG1	VX	0	<0.8	757 ± 56
		50	<0.8	
		100	1.6 ± 0.4	
		200	2.7 ± 0.2	
		400	5.1 ± 0.4	
	GA	200	7.0 ± 0.5	164 ± 22
GG2	GD	0	<0.8	3014 ± 561
		200	≈0.8	
		400	1.2 ± 0.2	
		1000	2.9 ± 0.2	
	GF	0	<0.8	1633 ± 279
		1000	2.3 ± 0.4	
	GB	0	<0.8	3778 ± 354
		1000	<0.8	
		3000	3.4 ± 0.3	

^a^ Undiluted enzyme = 20 µM; ^b^ Obtained by fitting a first order exponential equation. All data are based on two or three independent experiments. If an agent was degraded to levels below detection limit (≈3–5% of the original amount) within 4 min, then at least five half-lives have passed (100% × 0.5^5^ < 5%). It follows that the half-live in these cases is maximum 4 min/5 = 0.8 min; ^c^ Estimations were reached using the most diluted condition for each agent. n.d. = not determinable.

**Table 3:** Enzymatic degradation half-life of Novichok agents by a mixture of GG1 and GG2.

Enzyme(s)	Agent (25 mM)	Half-Life (min)	k_cat_ (s^−1^)
GG1/GG2	A230	<0.8 ^a^	>5.2
GG1/GG2	A232	2.5 ± 0.0 ^b^	3.7 ± 0.1
GG1/GG2	A234	t_1/2_ fast <0.8 ^a^	>5.2
	A234	t_1/2_ slow 76 ± 21 ^b^	0.1 ± 0.0

^a^ If an agent was degraded to levels below detection limits (≈3–5% of the original amount) within 4 min, then at least five half-lives have passed (100% × 0.5^5^ < 5%). It follows that the half-life in these cases is maximum 4 min/5 = 0.8 min. ^b^ Obtained by fitting a first order exponential equation.

## Data Availability

The data presented in this study are available on request from the corresponding author.

## Supplementary Materials

The following are available online at https://www.mdpi.com/article/10.3390/ijms22158152/s1.
