# Supplementary material for: Enzymatic Decontamination of G-Type, V-Type and Novichok Nerve Agents
Source: Int J Mol Sci. 2021 Jul 29;22(15):8152. doi: 10.3390/ijms22158152 (PMC8347808; doi:10.3390/ijms22158152)
Supplement: Supplementary file 1 [file ijms-22-08152-s001.zip › ijms-1309469-supplementary.pdf]

International Journal of
Molecular Sciences

Supplementary

Enzymatic Decontamination of G-type, V-type and Novichok Nerve Agents

Pauline Jacquet [1], Benjamin Rémy [1], Rowdy P.T. Bross [2], Marco van Grol [2], Floriane Gaucher [1], Eric Chabrière [3,4,*], Martijn. C. de Koning[2,*], David Daudé[1,*]

[1] Gene&GreenTK, 19–21 Boulevard Jean Moulin, 13005 Marseille, France; pauline.jacquet@gene-greentk.com (P.J.); benjamin.remy21@gmail.com (B.R.); floriane.gaucher@gene-greentk.com (F.G.)

[2] TNO department CBRN Protection, Lange Kleiweg 137, 2288GJ Rijswijk, The Netherlands; rowdy.bross@tno.nl (R.P.T.B.); marco.vangrol@tno.nl (M.v.G.)

[3] Institut Hospitalo-Universitaire (IHU) Méditerranée Infection, Aix-Marseille Université, 13005 Marseille, France

[4] Institut de Recherche pour le Développement (IRD), Assistance Publique Hôpitaux de Marseille (APHM), Unité Microbe Evolution Phylogénie et Infection (MEPHI), 13005 Marseille, France

* Correspondence: eric.chabriere@univ-amu.fr (E.C.); m.dekoning@tno.nl (M.C.d.K.); david.daude@gene-greentk.com(D.D.)

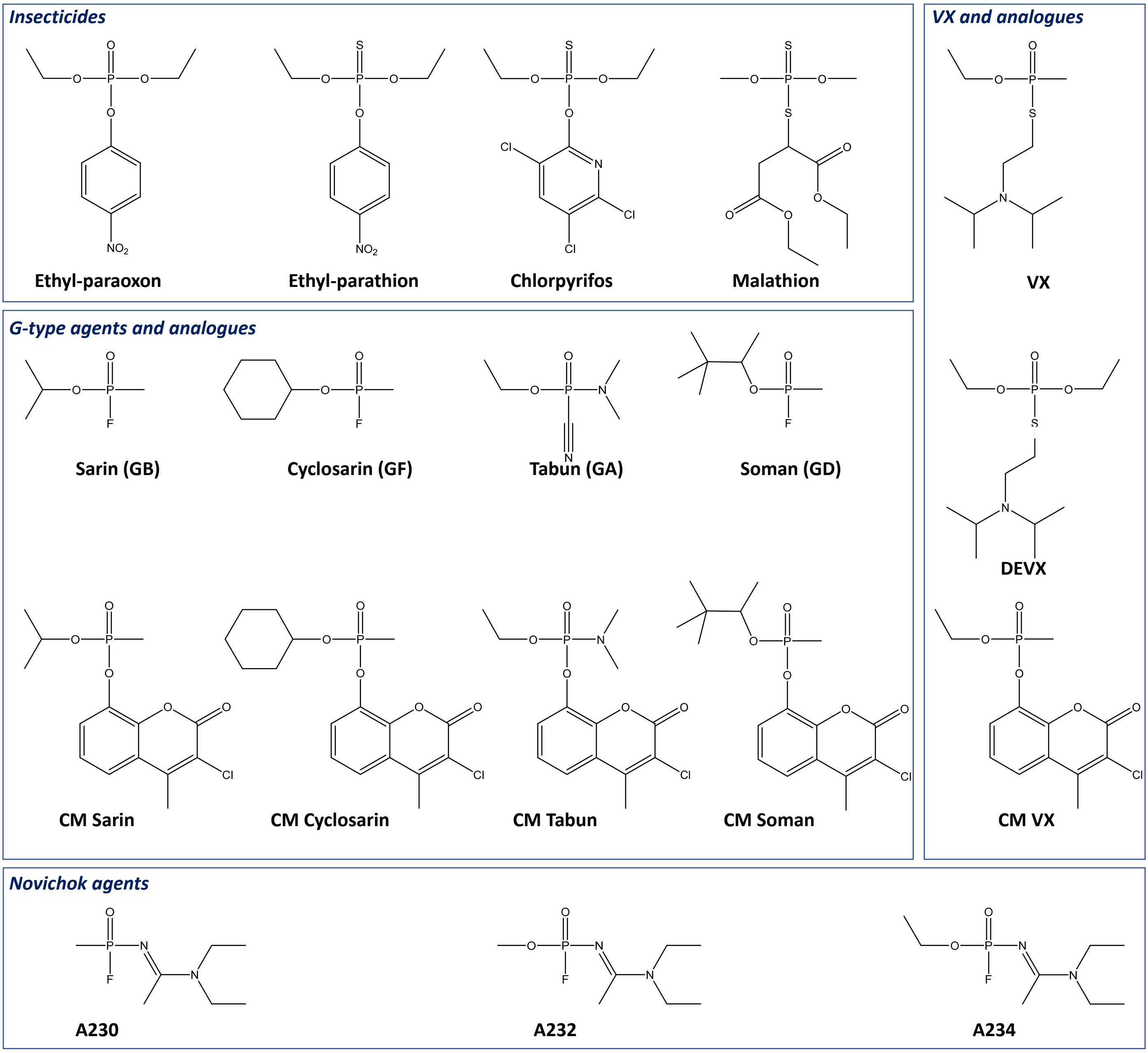

Supplementary Figure S1: Structures of OPs used in this study. Ethyl-paraoxon, a model insecticide in PTEs studies, was investigated together with three representative insecticides namely ethyl-parathion, chlorpyrifos and malathion. Four G-agents, namely sarin, cyclosarin, tabun and soman were considered together with their respective coumaric derivatives. VX was used as the reference V-agent with two surrogates CM VX and DEVX. Three Novichok agents A230, A232 and A234 were also studied.

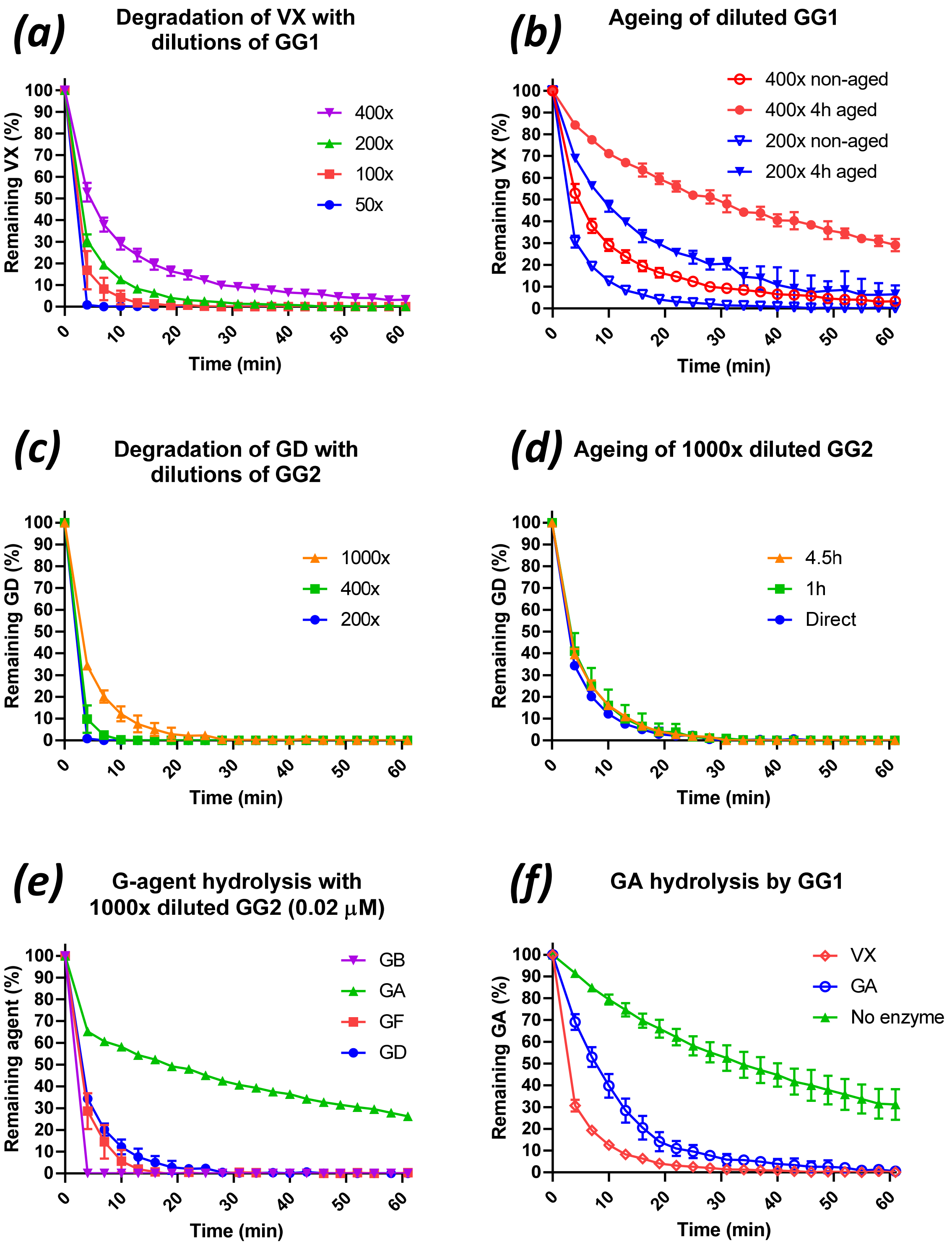

Supplementary Figure S2. Dilution and ageing of GG1 and GG2. Undiluted enzyme is 20 μM. (**a**) Evaluation of GG1 dilutions towards VX decontamination. (**b**) Impact of ageing on GG1 activity toward VX decontamination. (**c**) Evaluation of GG2 dilutions towards GD decontamination. (**d**) Impact of ageing on GG2 activity toward GD decontamination. (**e**) Evaluation 1000x diluted GG2 towards G-agents. (**f**) Comparison of GA and VX hydrolysis with GG1. All curves were assembled from at least 2 independent experiments.

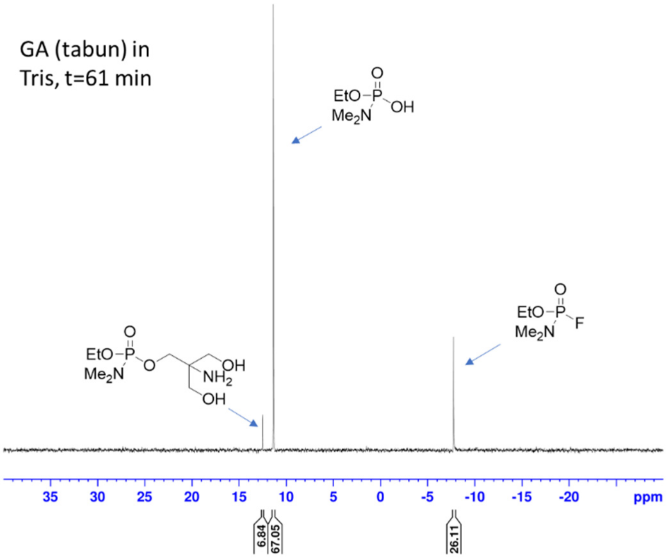

Supplementary Figure S3. ^{31}P NMR spectra of GA after 61 min of incubation in Tris buffer (200 mM) time point of positive control. −8 ppm: GA, 13 ppm: tabun-Tris adduct, 11ppm: Ethyl *N*,*N*-dimethylphosphoramidate (hydrolysis product).

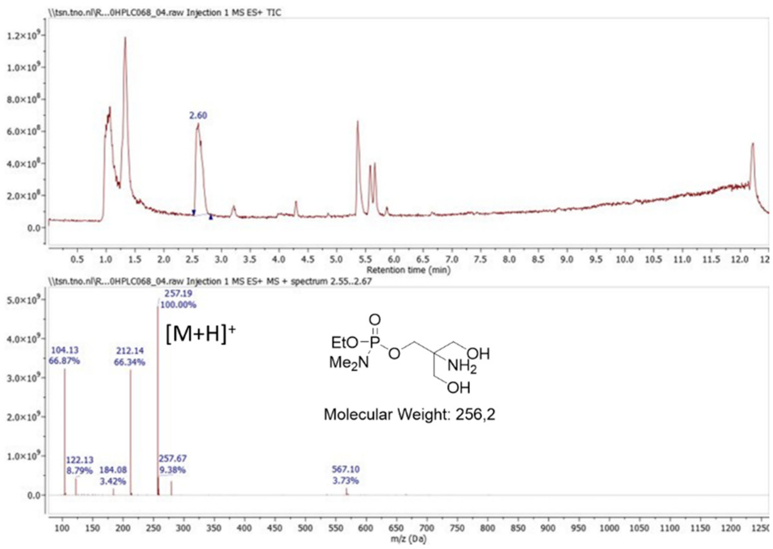

Supplementary Figure S4. LC-MS trace and MS spectrum of GA control mixture (measured after standing overnight). The peak at 2.6 min is the GA-TRIS adduct ($[M+H]^+$ = 257).

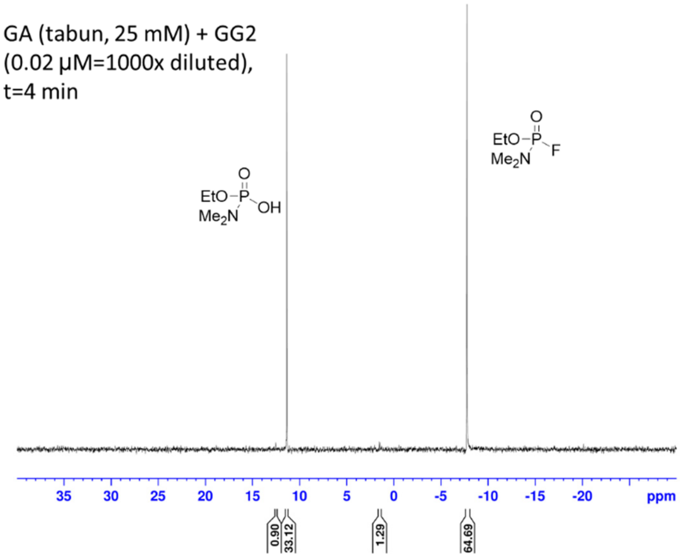

Supplementary Figure S5. ^{31}P NMR spectra of GA after four minutes of incubation in Tris buffer (200 mM) with 1000 dilution of GG2 (0.02 µM).

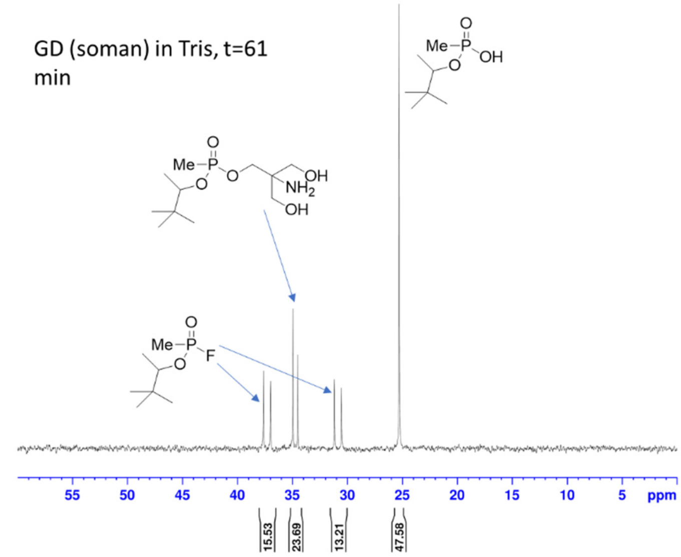

Supplementary Figure S6. ^{31}P NMR spectra of GD (control without enzyme) after 61 min of incubation in Tris buffer. Besides residual GD and hydrolysis product, there is a third product (TRIS-GD adduct).

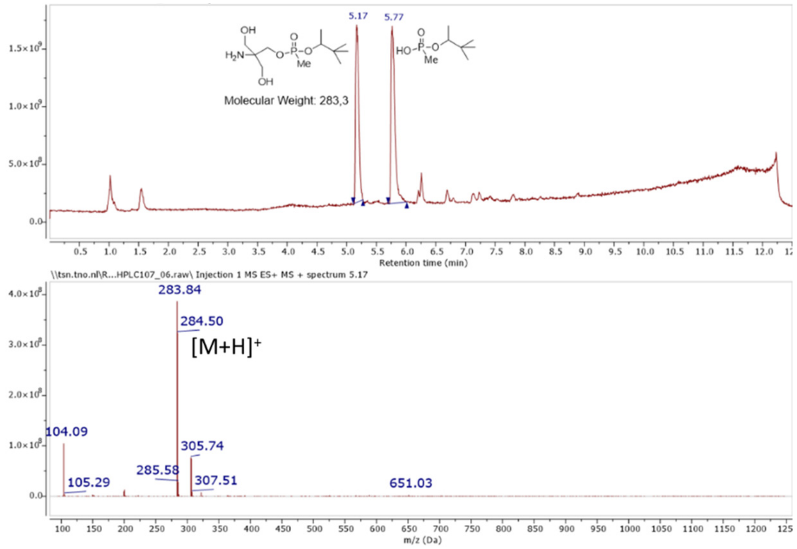

Supplementary Figure S7. LC-MS trace and MS spectrum of GD control mixture (measured after standing overnight). The peak at 5.17 min is the GD-TRIS adduct ($[M+H]^+$ = 284)

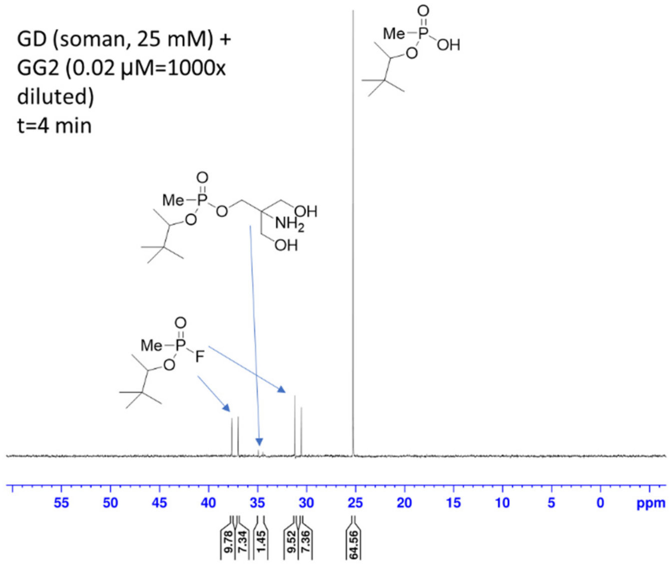

Supplementary Figure S8. ^{31}P NMR spectra of GD after four minutes of incubation in Tris buffer (200 mM) with 1000 dilution of GG2 (0.02 µM).

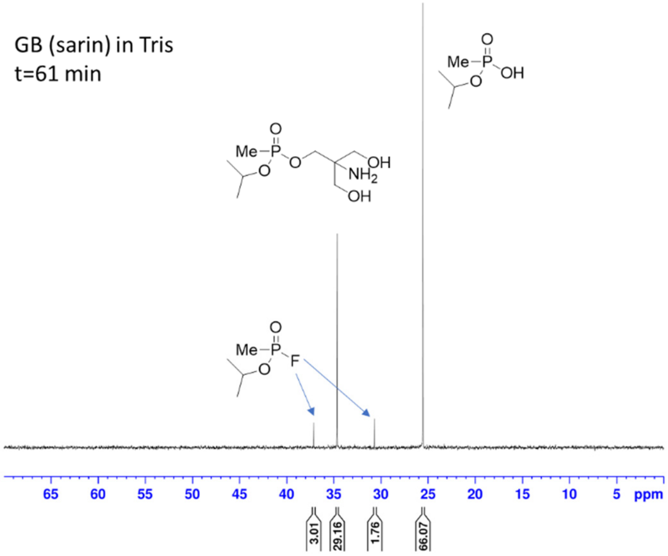

Supplementary Figure S9. ^{31}P NMR spectra of GB (control without enzyme) after 61 min of incubation in Tris buffer. 31/37 ppm: sarin, 35 ppm: adduct, 25 ppm: isopropyl methylphosponate (IMPA).

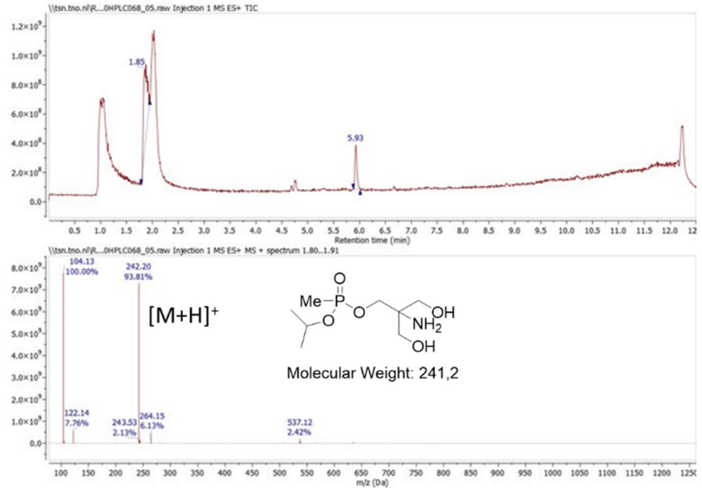

Supplementary Figure S10. LC-MS trace and MS spectrum of GB control mixture (measured after standing overnight). The peak at 1.85 min is the GB-TRIS adduct ($[M+H]^+ = 242$).

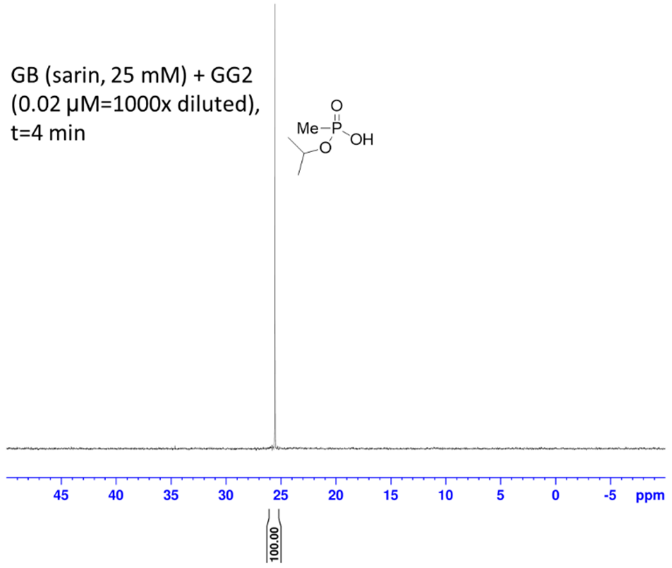

Supplementary Figure S11. ^{31}P NMR spectra of GB after four minutes of incubation in Tris buffer (200 mM) with 1000 dilution of GG2 (0.02 µM). 25 ppm: isopropyl methylphosponate (IMPA).

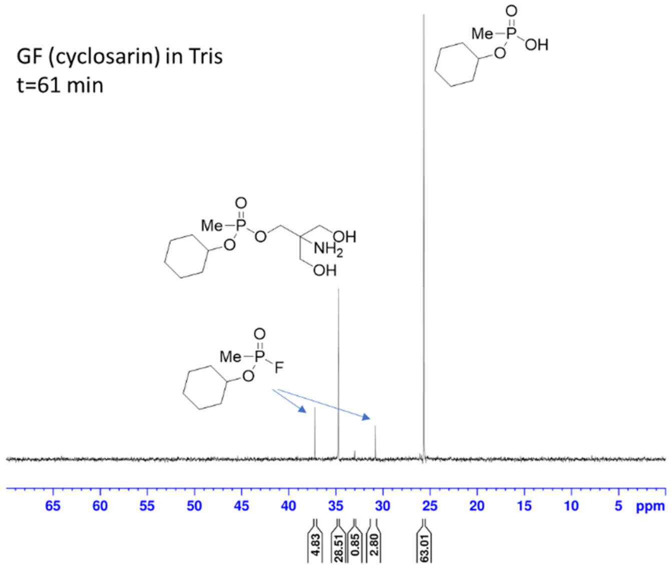

Supplementary Figure S12. ^{31}P NMR spectra of GF (control without enzyme) after 61 min of incubation in Tris buffer. 31/37 ppm: GF, 35 ppm: adduct, 26 ppm: cyclohexyl methylphosphonate (hydrolysis product).

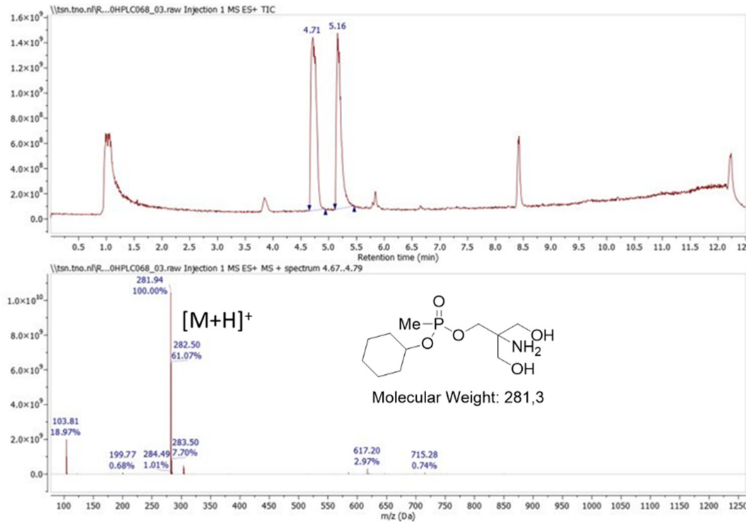

Supplementary Figure S13. LC-MS trace and MS spectrum of GF control mixture (measured after standing overnight). The peak at 4.71 min is the GF-TRIS adduct ($[M+H]^+$ = 282).

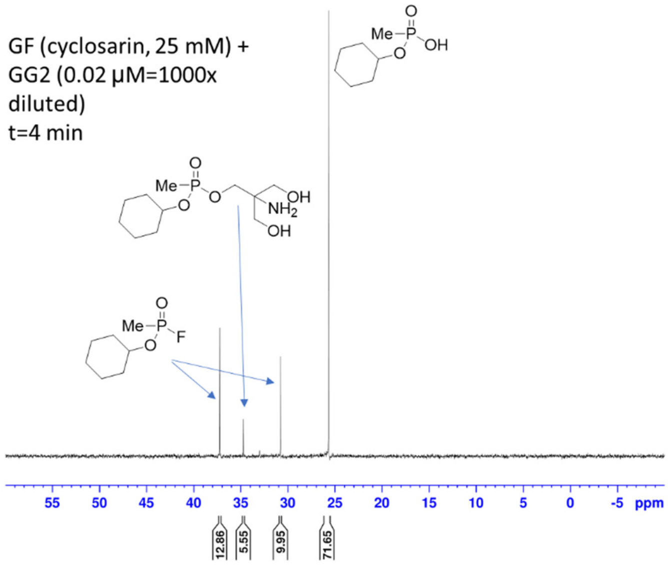

Supplementary Figure S14. ^{31}P NMR spectra of GF after four minutes of incubation in Tris buffer (200 mM) with 1000 dilution of GG2 (0.02 µM). 26 ppm: cyclohexyl methylphosphonate (hydrolysis product).

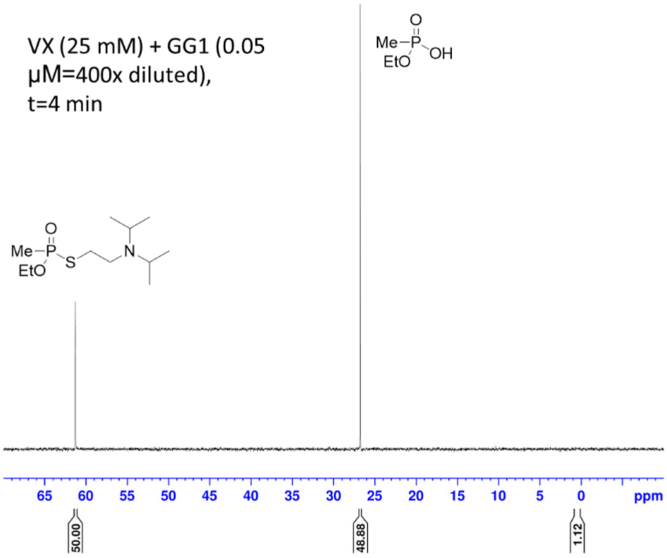

Supplementary Figure S15. ^{31}P NMR spectra of VX after four minutes of incubation in Tris buffer (200 mM) with 400 dilution of GG1 (0.05 μM).

In silico analysis of GG1 and GG2 interaction with A230 enantiomers

Docking was realized with AutoDockTools (1.5.6 version) software. PTE wild-type was used as model (PDB 1EZ2) to add GG1 and GG2 mutations using WinCoot (0.8.3 version) software. Three-dimensional structures of A230 (R) and (S) enantiomers were prepared with MarvinJS (https://marvinjs-demo.chemaxon.com/latest/demo.html, ChemAxon). On AutoDockTools, Gasteiger charges were added to GG1 and GG2, and docking was performed using Lamarckian genetic algorithm. Best configurations were selected with the lowest estimated free energy of binding (kcal/mol) and the most relevant positioning within the active site. Docking results were visualized using PyMOL software.

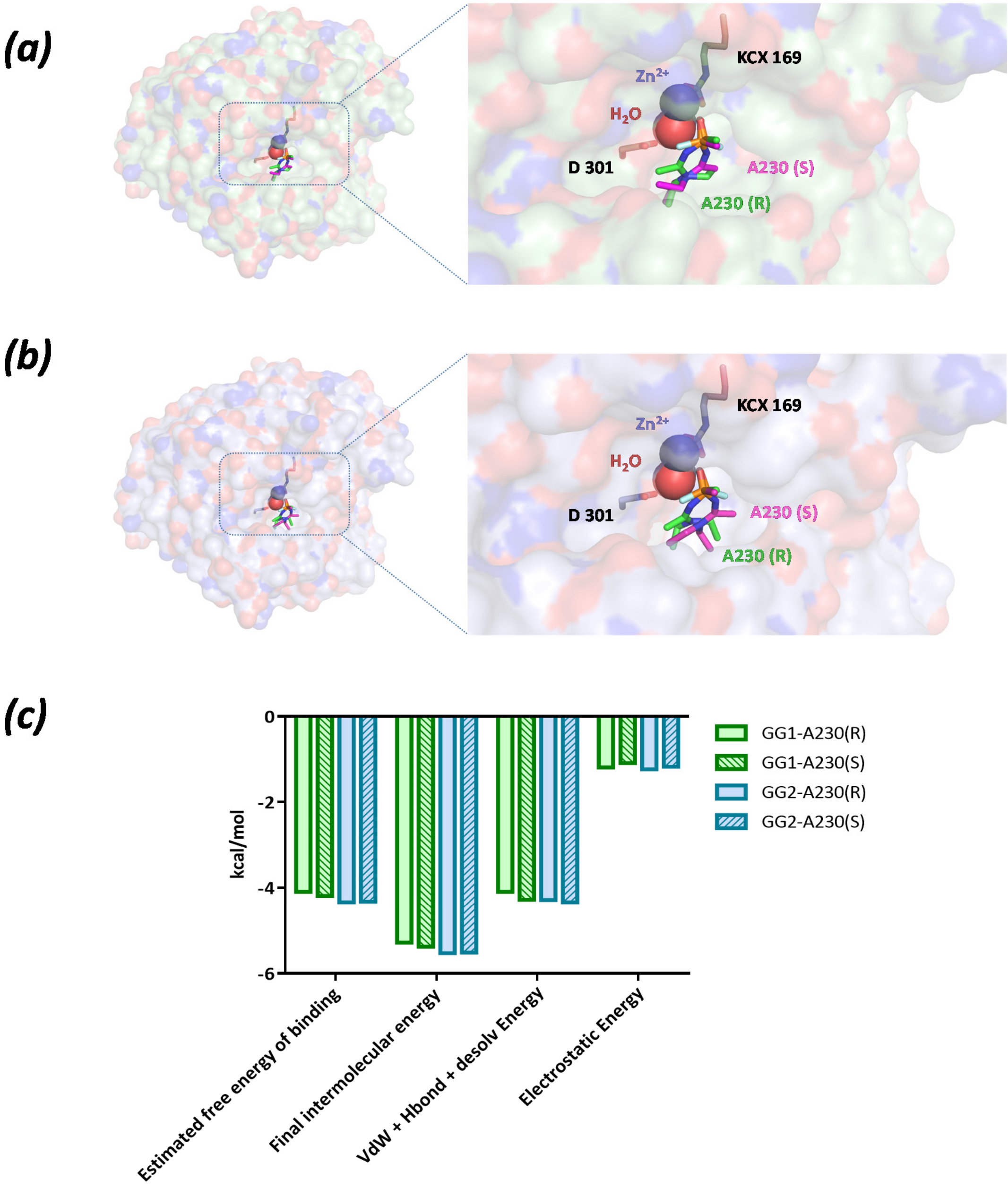

Supplementary Figure S16. Docking of Novichok A230 (R) and (S) enantiomers with GG1 and GG2, created using PTE wild-type as model (PDB 1EZ2). (**a**) Docking of GG1 with A230 (R) and (S). (**b**) Docking of GG2 with A230 (R) and (S). Aspartic acid 301 (D 301) and carboxylated lysine 169 (KCX 169) are active site's residues. (**c**) Estimated energies obtained for best conformations.

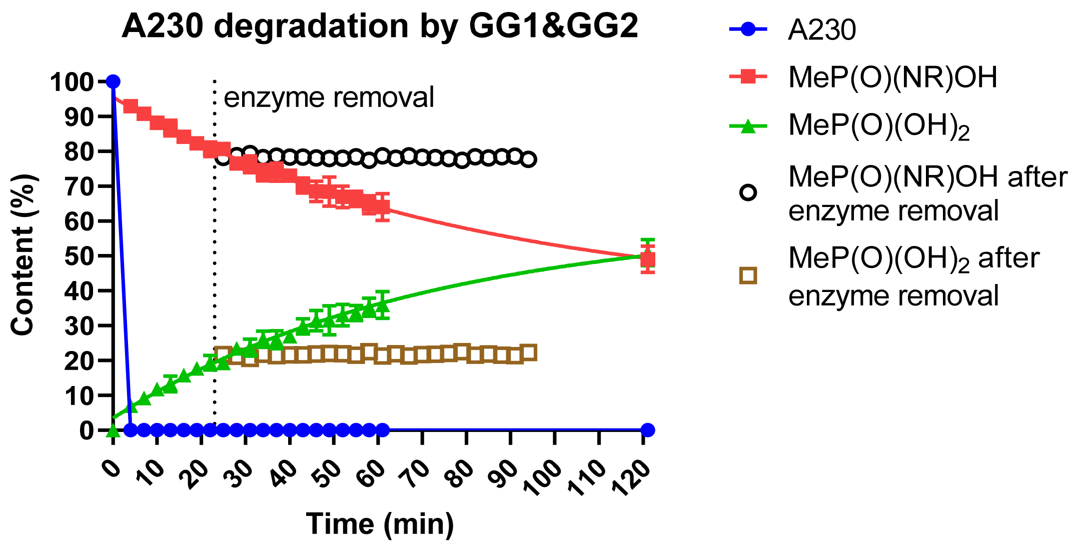

Supplementary Figure S17. Degradation of A230 by a mixture of the enzymes GG1 & GG2 leads to nearly instantaneous complete hydrolysis of A230 and the concomitant formation of MeP(O)(NR)OH, which in turn is hydrolyzed into MeP(O)$(OH)_2$. This graph shows the curve obtained after filtration of the reaction mixture over a 10 kD MW cut-off filter at t=22 min (open circles/squares), in comparison with the reaction in the presence of enzyme. After filtration, the concentrations of both hydrolysis products remain constant, indicating that the secondary conversion of MeP(O)(NR)OH into methylphosphonic acid is enzymatically catalyzed. This experiment was carried out in duplicate (error bars are too small to show). We have further included the 120 min time point that was omitted in Figure 3c.
